# Indigenous communities and climate-related hazards: A protocol for a systematic review

**DOI:** 10.1016/j.mex.2023.102514

**Published:** 2023-12-09

**Authors:** Cesar Cervantes Benavides, Hamed Seddighi Khavidak, Rónán Mc Dermott, Caspar van den Berg

**Affiliations:** a Campus Fryslân, Global and Local Governance — Faculty Board, University of Groningen, Wirdumerdijk 34, 8911 CE Leeuwarden, the Netherlands.; b Faculty of Behavioural and Social Sciences, University of Groningen, Grote Kruisstraat 2/1 9712 TS Groningen The Netherlands.

**Keywords:** Ethnicity, Climate change, Hazards, Indigenous communities, Risk perception, Indigenous communities and climate-related hazards: a protocolfor a systematic review

## Abstract

As reported by World Bank figures, in 2020, there were about 476 million indigenous people living in more than 90 countries around the world. They represented more than 6 % of the world's population. Approximately 15 % of these indigenous people lived in conditions of extreme poverty, facing economic disparity and chronic vulnerability [36]. This review analyzes the risks faced by indigenous communities due to climate change and their perception of risk. Hazards are identified in different regions, considering direct and indirect impacts on territories, resources and ways of life.

## Graphical abstract

**Figure:**
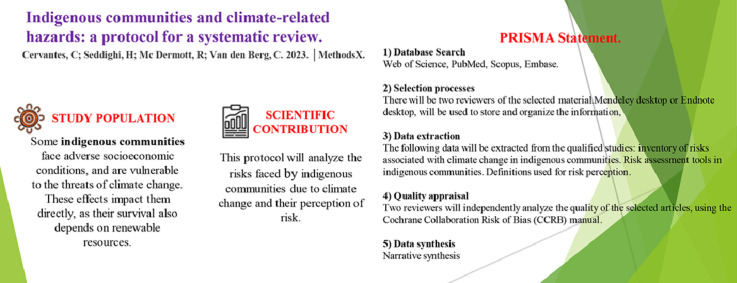

Specifications table

**Table:** 

Subject area:	Environmental Science
More specific subject area:	Climate Change
Name of your protocol:	Indigenous communities and climate-related hazards: a protocol for a systematic review.
Reagents/tools:	Preferred Reporting Items for Systematic Reviews and Meta-Analyses guidelines to develop the protocol for this systematic review [1]. The Cochrane manual of systematic reviews of interventions will also be consulted [2].
Experimental design:	The selection and audit of studies for this review will be based on clearly established inclusion and exclusion criteria, in order to finally evaluate and communicate the results obtained
Trial registration:	Not applicable
Ethics:	Not applicable, since this is a systematic review protocol of the reported scientific literature.
Value of the Protocol:	1. Some Indigenous communities live in conditions of extreme poverty, face economic disparities, chronic vulnerability, and are often geographically located in areas with complex climates. As a result, with higher rates of mortality, morbidity and marginalization, they are particularly vulnerable to the adverse effects of climate change. 2. Threats caused by climate change have a direct impact on indigenous populations because their survival also depends on renewable resources [3] and on mitigation and adaptation measures [4]. However, most of these communities lack mitigation plans to cope with climate change risks [5]. 3. Therefore, trying to classify and define the types of hazards faced by indigenous groups represents the first step in diagnosing the level of exposure to the hazard, and lays the foundation for the design of public policies or strategies to prevent or mitigate the risk.

## Description of protocol

**Indigenous communities and climate-related hazards: a protocol for a systematic review.**

## Introduction

According to international classification perspectives recognized by the International Labor Organization (ILO), indigenous, aboriginal, and tribal peoples are those population groups in different geographical regions surviving periods of colonization and domination by foreign forces [3]. Their social organization is determined by the existence of ancestors and a common history.

Relative to anthropology, indigenous peoples are distinguished by a distinct ethnicity in terms of jurisdiction, territory, organisms, and cognitive and spiritual aspects [6]. That is to say; ethnicity itself draws traditions, shared rituals, consolidated social institutions, and cultural traits (language, gastronomy, music, dance, and spirituality among other elements) [7].

As reported by World Bank figures, in 2020, there were about 476 million indigenous people living in more than 90 countries around the world. They represented more than 6 % of the world's population. Approximately 15 % of these indigenous people lived in conditions of extreme poverty, facing economic disparity and chronic vulnerability [8]. This economic inequality and ongoing vulnerability translate into a lower life expectancy of up to 20 years compared to non-indigenous people (The International Work Group for Indigenous Affairs [9]). As a result, indigenous people are among the most disadvantaged and extremely vulnerable to environmental risks [10].

Moreover, some of these minority groups are geographically located in arid or semi-arid zones [11,12]. These lands cover approximately one-third (48,350,000 km2) of the earth's surface. It is estimated that 13 % of the world's population resides in these zones [13]. Precipitation tends to be scarce and below the maximum potential annual evaporation, which expresses an increase in daily temperature [14]. Because of this, some indigenous communities in arid zones have already been involved in struggles for water.

Water issues are usually related to three aspects: quantity, quality, and availability of water [15]. Thus, indigenous groups settled in arid areas, at the mercy of chronic vulnerability, are the ideal scenario for social dislocations to multiply and poverty to become almost insurmountable [12]

In addition, with complicated geography, these indigenous groups face one of the greatest challenges: climate change [[16], [17]]. Defined as a natural phenomenon comprising a long-term change in temperatures and weather patterns, but which, due to anthropogenic damage such as the burning of fossil fuels, has generated a greenhouse effect. So specifically in recent years the highest temperatures in almost two million years have been recorded [18].

However, the effect of climate change is not only the increase in temperature but because the Earth is a system, various adverse factors on natural resources, biogeochemical cycles, and global public health are multiplied [19].

According to the United Nations Intergovernmental Panel on Climate Change (IPCC), the effects of climate change will have the greatest impact on indigenous populations because they have the highest rates of mortality, morbidity, and marginality [20,21].

The hazards brought about by climate change have a direct impact on indigenous populations due to the fact that their survival also depends plainly and simply on renewable resources [22], and mitigation and adaptation measures to its effects [4]. Nevertheless, most of these communities lack mitigation plans to cope with climate change risks [5].

On the other hand, in recent years there have been several disputes between indigenous communities versus federal governments or corporations. The struggle for legal recognition of their territories and natural resources has been the main trigger for these conflicts [23,24]. In addition, indigenous struggles are also related to the claim for the almost irreparable anthropogenic damage that various corporations and governments have perpetuated without considering that nature is where some indigenous groups celebrate the rituals or customs that signify and dignify them as a community [25].

Unfortunately, despite the legislative efforts of the United Nations (UN) in terms of social justice for indigenous peoples, the 21st century reflects a negative picture [26]. A constant indifference prevents them from being part of the formal economy and from being consulted in political decision-making processes [27].

This shows that achieving the goals of the 2030 Agenda for Sustainable Development will not be an easy task.

### Climate change hazards

Potential hazards caused by climate change can be defined as the probability that weather changes or phenomena will have a prolonged effect on specific areas such as crops, workplaces, sacred sites, etc., or on the well-being and health of populations and their territories (United Nations Office on Drugs and Crime [28]).

There are various ways of classifying hazards, whether by meteorological, hydro meteorological or climatological aspects, and they are also defined by their geographic impact, spectrum, intensity and duration [28].

According to Conde [29], there are four categories of hazards to climate change; the first includes risks to basic resources (water, agriculture and food); the second includes social effects (health, possible mass migrations, challenges to human rights); the third includes political effects (risk of an increase in "failed states''); and finally, economic-political effects (economic inequalities, ``energy security'', struggle for access to energy resources).

### Aim of review

In this context, this systematic review aims to know, document, and understand the various manifestations and risks caused by climate patterns, with special emphasis on global and local cases involving indigenous communities.

Similarly, it is necessary to know how different authors approach the concept of risk perception or its synonyms, as well as the instruments they use to measure it.

The urgency lies in the fact that the Intergovernmental Panel on Climate Change (Intergovernmental Panel on Climate Change [30]), considers the study of risk perception: "understood as the subjective assessment that individuals make of the probability and severity of an adverse event", is important for the survival of future generations, being fundamental the inclusion of vulnerable indigenous groups in these studies [31].

Hence, the "Hyogo Framework for Action 2005–2015: Building the Resilience of Nations and Communities to Disasters" states that climate change should be considered a visible threat to the future of the world. Therefore, risk planning as one of the first steps to address the challenges posed by climate change [32].

Likewise, according to Wachinger et al. [33], it is said that the individual's personal experience, and trust in studies or mitigation programs generated by experts or scientific authorities, are important factors in the perception of natural hazards in the face of climate change.

In addition, understanding how indigenous communities perceive and construct their paradigms of life and risk can provide information on how these cultures preserve their social practices and adapt to climate change. This knowledge is vital to prevent and mitigate conflicts and environmental damage in areas with an indigenous presence.

Finally, according to Uriel Beck [34], as the danger becomes increasingly prominent, some researchers choose to present their projects in a risk framework.  This has given rise to productive international risk research networks, such as within the European Sociological Association (Research Network 22, RN22) and the International Sociological Association (Thematic Group 04, TG04) [35].

**Research question:**

What are the specific hazards and risks faced by ethnic communities in different regions of the world due to climate change, and how do these communities perceive and assess these risks?

**Secondary objectives:**

• To classify the hazards to which ethnic communities are exposed due to climate change.
• To describe the diverse conceptions of risk perception within indigenous communities in relation to climate change.
• To detect what type of instruments are used to measure the perception of environmental risk as reported in the scientific literature.
• To identify information and actions that could be useful to address climate change risks.

## Method

Preferred Reporting Items for Systematic Reviews and Meta-Analyses guidelines to develop the protocol for this systematic review [1]. The Cochrane manual of systematic reviews of interventions will also be consulted, since the instruction provides a safe handling of the available clinical and social information or empirical evidence, thus achieving the identification, collation and synthesis, with a lower risk of bias, i.e.; it will serve to define the research questions by means of the elements Population, Interventions, Comparators and Outcomes [2].

The selection and audit of studies for this review will be based on clearly established inclusion and exclusion criteria, in order to finally evaluate and communicate the results obtained.

## Eligibility

### Inclusion criteria

#### Type of participants

The review will include indigenous people according to the criteria for the identification of indigenous peoples in the United Nations Declaration on the Rights of Indigenous Peoples, 2007, understood as those who have inherited and practice unique forms of spiritual, cultural, social and economic relationships in their territories. They are also peoples who often have a special relationship with the land and natural resources [24].

#### Context/setting

Regions with greater susceptibility and exposure to the adverse effects of climate change, making them particularly vulnerable. Areas are prone to hazards and conflicts arising from climate change, such as rising temperatures, changes in precipitation patterns, extreme weather events, and sea level rise.

#### Type of interventions

The type of intervention considered in this study is observational/descriptive intervention.

#### Type of studies

Descriptive studies that analyze the various hazards associated with climate change in indigenous peoples will be included in the review. Qualitative or quantitative studies with an experimental or quasi-experimental design.

#### Types of publication

The study will include theoretical research papers published in peer-reviewed scientific journals or conference proceedings that are accompanied by full-length peer-reviewed articles.

#### Outcome measures

The following information will be obtained from the items:

• Inventory of risks associated with climate change in ethnic communities.
• Risk assessment tools in indigenous communities.
• Definitions used for risk perception.

### Exclusion criteria

#### Type of participants

Participants who are not considered an indigenous population as previously stated.

#### Context/setting

Studies conducted in urban areas will be excluded.

#### Type of interventions

Studies that do not have a clear and detailed observational intervention will be excluded.

#### Type of studies

Theoretical development studies without empirical observation, which are not scientific studies or which have not been peer-reviewed, will be excluded.

### Search strategy

We will use electronic databases for the exhaustive literature search, such as Web of Science, PubMed, Scopus, Embase, and Ebsco databases, to identify records that met the above inclusion criteria.

During the initial literature search, different keywords were identified for the systematic search. The main search terms are: “indignity”, “ethnicity”, “hazard”, “threat”, “climate” and “change”.

The keyword combinations related to population, interest and context and the draft search strategy in the selected databases are presented in the supplementary material. The search terms will be combined with appropriate Boolean operators and the search will be based on titles, abstracts and keywords.

### Selection processes

There will be two reviewers of the selected material. The bibliographic manager: Mendeley desktop or Endnote desktop, will be used to store and organize the information, to later eliminate duplicate material.

Both abstracts and full texts will be reviewed to select the material within this systematic review. Each reviewer should examine and classify the material separately, but taking into consideration the previously agreed inclusion and exclusion criteria, and according to the type of intervention, the sample population and the results recorded.

In the event of any conflict of interest or ethical dilemma during the review, and which causes the two reviewers not to reach a common agreement with respect to the material analyzed, a third reviewer will have the final decision.

### Data extraction

Data retrieval will be performed individually by two reviewers and agreement will be reached through conversation.

In the event that the investigators are unable to resolve disagreements internally, there is the option of using an impartial and neutral third reviewer. This third party will conduct a review of the disputed studies and make an independent decision to resolve the disagreement.

The following data will be extracted from the qualified studies:

• Inventory of risks associated with climate change in indigenous communities.
• Risk assessment tools in indigenous communities.
• Definitions used for risk perception.

### Quality appraisal

Two reviewers will independently analyze the quality of the selected articles, using the Cochrane Collaboration Risk of Bias (CCRB) manual, which allows measuring the risk of bias in randomly selected materials under 5 dimensions: selection bias, performance bias, detection bias, attrition bias, and information bias [2].

Likewise, the Critical Appraisal Skills Program checklist recommended by the Cochrane Collaboration for qualitative literature will be used to measure the risk of bias in qualitative studies. This checklist consists of 10 questions (nine questions addressing quality and one addressing "value”) [36].

### Data synthesis

Taking as a reference how diverse the studies to be analyzed can be, a narrative synthesis will be presented, and not a statistical aggregation of the data. Narrative synthesis brings together the main results or findings within the systematized analysis and thereby generates the construction of the state of the art [37].

In addition, the narrative synthesis is not only responsible for characterizing or summarizing the selected studies, it also allows to evaluate or establish the differences and similarities between the analyzed data [38].

Therefore, four steps will be followed to perform the narrative synthesis [39]:
Develop a theory about how the intervention works, why, and for whom.
Developing a preliminary synthesis of findings from the included studies.
Explore relationships in the data.
Assess the robustness of the synthesis.

During the first step of the review, the risks associated with climate change in indigenous communities and the definitions and approach to risk perception will be detailed. Subsequently, the various risk assessment tools used in indigenous communities will be described. The third step will consist of synthesizing the definitions used for risk perception in the context of indigenous communities.

In the last step, the aim is to describe the perception of risk in indigenous communities and its relationship with social vulnerability, identifying possible obstacles for the development of policies aimed at the protection of natural resources in these communities.

## Additional information

From the systematic review and analysis of the existing literature, the relevance and urgency of investigating the risks associated with climate change in indigenous communities will emerge.

These communities, face specific challenges and hazards due to their dependence on natural resources and their cultural and spiritual connection to the environment. In addition, the economic inequality and social marginalization to which they are exposed make them particularly vulnerable to the impacts of climate change.

The proposed study aims to understand and document the various ways in which climate change affects indigenous communities, as well as to assess the associated risks and explore risk perceptions from the perspective of these communities.

This systematic review will contribute to generating knowledge and awareness of the importance of addressing the risks and protecting the rights of ethnic communities in the context of climate change. It will also inform the design of adaptation and mitigation policies and strategies that are culturally appropriate and that promote the resilience and well-being of these communities, since risk and hazards should be considered an omnipresent variable in society, a growing and changing potentiality, waiting to be revealed when a hazardous event occurs [40].

Finally, in addressing the hazards posed by climate change, this systematic review will argue that the multiplier effects of climate change are a matter of international human security, and that social justice itself, calls for a rethinking of the current role of environmental justice in the field of human rights and, in the displacement, migration and forced relocation of affected indigenous communities [41].

## CRediT authorship contribution statement

**Cesar Cervantes Benavides:** Conceptualization, Methodology, Writing – original draft, Resources. **Hamed Seddighi Khavidak:** Validation, Methodology, Supervision, Writing – review & editing. **Rónán Mc Dermott:** Validation, Supervision, Writing – review & editing. **Caspar van den Berg:** Supervision, Writing – review & editing.

## Declaration of Competing Interest

The authors declare that they have no known competing financial interests or personal relationships that could have appeared to influence the work reported in this paper.

## Data Availability

Data will be made available on request.

## Appendix Supplementary materials

Image, application 1
