# Supplementary material for: Indigenous communities and climate-related hazards: A protocol for a systematic review
Source: MethodsX. 2023 Dec 9;12:102514. doi: 10.1016/j.mex.2023.102514 (PMC10727928; doi:10.1016/j.mex.2023.102514)
Supplement: Supplementary file 1 [file mmc1.docx]

**Table S1.** Database search strategy.

| **Data base** | **Search strategy** | **Date** |
| --- | --- | --- |
| PubMed | ((indigenous) OR (ethnicity) OR (native populations)) AND ((hazard) OR (threats)) AND ((climate change) OR (global warming) OR (climate variability)) Filters: English | Jul 28th, 2023.  279 articles. |
| Scopus | KEY ( ( ( indigenous ) OR ( ethnicity ) OR ( native AND populations ) ) AND ( ( hazard ) OR ( threats ) ) AND ( ( climate AND change ) OR ( global AND warming ) OR ( climate AND variability ) ) ) AND ( LIMIT-TO ( DOCTYPE , "ar" ) ) | Jul 28th, 2023.  789 articles. |
| Web of Science | ((indigenous) OR (ethnicity) OR (native populations)) AND ((hazard) OR (threats)) AND ((climate change) OR (global warming) OR (climate variability)) Refined By: Document Types: Article, Languages: English | Jul 28th, 2023.  1011 articles. |
| Embase | ('indigenous'/exp OR indigenous OR 'ethnicity'/exp OR ethnicity OR 'native populations' OR (('native'/exp OR native) AND populations)) AND ('hazard'/exp OR hazard OR threats) AND ('climate change'/exp OR 'climate change' OR (('climate'/exp OR climate) AND ('change'/exp OR change)) OR 'global warming'/exp OR 'global warming' OR (('global'/exp OR global) AND ('warming'/exp OR warming)) OR 'climate variability'/exp OR 'climate variability' OR (('climate'/exp OR climate) AND ('variability'/exp OR variability))) AND [english]/lim | Jul 28th, 2023.  166 articles. |
